# XLPM: efficient algorithm for the analysis of protein-protein contacts using chemical cross-linking mass spectrometry

**DOI:** 10.1186/1471-2105-15-S11-S16

**Published:** 2014-10-21

**Authors:** Mihir Jaiswal, Nathaniel Mark Crabtree, Michael A Bauer, Roger Hall, Kevin D Raney, Boris L Zybailov

**Affiliations:** 1 Department of Biochemistry and Molecular Biology, University of Arkansas for Medical Sciences, Little Rock, AR 72205, USA; 2 UALR/UAMS Joint Bioinformatics Programme, University of Arkansas Little Rock, Little Rock, AR 72205, USA; 3 Department of Biomedical Informatics, University of Arkansas for Medical Sciences, Little Rock, AR 72205, USA; 4 Myeloma Institute for Research and Therapy, University of Arkansas for Medical Sciences, Little Rock, AR 72205, USA

**Keywords:** chemical cross-linking mass spectrometry, protein-protein interactions, peptide fragmentation, computational proteomics

## Abstract

**Background:**

Chemical cross-linking is used for protein-protein contacts mapping and for structural analysis. One of the difficulties in cross-linking studies is the analysis of mass-spectrometry data and the assignment of the site of cross-link incorporation. The difficulties are due to higher charges of fragment ions, and to the overall low-abundance of cross-link species in the background of linear peptides. Cross-linkers non-specific at one end, such as photo-inducible diazirines, may complicate the analysis further. In this report, we design and validate a novel cross-linked peptide mapping algorithm (XLPM) and compare it to StavroX, which is currently one of the best algorithms in this class.

**Results:**

We have designed a novel cross-link search algorithm -XLPM - and implemented it both as an online tool and as a downloadable archive of scripts. We designed a filter based on an observation that observation of a b-ion implies observation of a complimentary y-ion with high probability (b-y filter). We validated the b-y filter on the set of linear peptides from NIST library, and demonstrate that it is an effective way to find high-quality mass spectra. Next, we generated cross-linked data from an ssDNA binding protein, Rim1with a specific cross-linker disuccinimidyl suberate, and a semi-specific cross-linker NHS-Diazirine, followed by analysis of the cross-linked products by nanoLC-LTQ-Orbitrap mass spectrometry. The cross-linked data were searched by XLPM and StavroX and the performance of the two algorithms was compared. The cross-links were mapped to the X-ray structure of Rim1 tetramer. Analysis of the mixture of NHS-Diazirine cross-linked ^15^N and ^14^N-labeled Rim1 tetramers yielded ^15^N-labeled to ^14^N-labeled cross-linked peptide pairs, corresponding to C-terminus-to-N-terminus cross-linking, demonstrating interaction between different two Rim1 tetramers. Both XLPM and StavroX were successful in identification of this interaction, with XLPM leading to a better annotation of higher-charged fragments. We also put forward a new method of estimating specificity and sensitivity of identification of a cross-linked residue in the case of a non-specific cross-linker.

**Conclusions:**

The novel cross-link mapping algorithm, XLPM, considerably improves the speed and accuracy of the analysis compared to other methods. The quality selection filter based on b-to-y ions ratio proved to be an effective way to select high quality cross-linked spectra.

## Background

Mapping of protein-protein interactions (PPIs) is an important, yet challenging analytical problem. This is also a potential clinical need, as having a detailed PPI information may aid in finding novel targets for therapeutic intervention [1]. Thus far, mainstream methods of PPI mapping include affinity-purification-mass-spectrometry (AP-MS)[2], yeast-two-hybrid (Y2H)[3], or mammalian-two hybrid methods. PPI information obtained from these experiments is deposited into online databases, such as BioGRID [4]. However, it takes a lot of effort to map the full interactome using these approaches. Also, as researches are starting to ask questions about interactome dynamics [5], we need faster and more effecting ways to map PPI networks. We suggest that PPI sampling using chemical cross-linking is an attractive alternative to AP-MS and Y2H methods.

In our recent review article on the subject, we presented a case for the use of short-length cross-linkers of broad specificity for the purpose of sampling PPIs and discussed existing challenges and limitations [6]. We argued that a short, broadly specific cross-linker could yield a more comprehensive interactome analysis and, in some cases, the mapping of individual PPI interfaces. The main difficulties include identification of cross-linked peptides in the background of non-cross-linked species and also, discriminating between inter- and intra-protein cross-links and assessing the false positive rate of cross-linked site identification. The other technical problem is the computational expense of allowing for non-specific cross-linking (i.e. cross-linked to any amino acid). We proposed the following steps to overcome these difficulties. Step 1 - starting at the scale of few proteins, develop an efficient filtering and scoring system to analyze cross-linking data, allowing for non-specific cross-linking at one end. Step 2 - expand to the large scale removing all specificity requirements. In this publication we complete the Step 1 and build a foundation to move onto Step 2.

For a scale of few proteins, it is easy to design a cross-link search algorithm, when one is relying on a database consisting of pair-wise combinations of peptides from interacting proteins. Next, from this database the list of possible cross-linked precursors is prepared. There are many software tools that use this approach: CLPM [7], xComb [8], GPMAW [9], X!Link [10], StavroX [11], MassMatrix [12]. However, all of them perform rather poorly when non-specificity is allowed. Also, not all of those algorithms take a full advantage of MS/MS spectra for defining the position of the cross-linked amino acid residues.

In this paper, we present a new algorithm, XLPM, which uses a novel method to allow effective use of the tandem MS information for the cross-link analysis without dramatically affecting the speed when non-specificity is allowed. We used StavroX as a benchmark to evaluate the XLPM performance, because, in our hands, StavroX produced the most robust and reliable data amongst all the programs we evaluated. We used the photo labile diazirine cross-linker SDA [13] on a Rim1 tetramer to evaluate the XLPM. Rim1 is a single strand DNA binding protein localizing to yeast mitochondria. Our data suggest a previously unreported tetramer-to-tetramer interaction. The functional analysis of this interaction and its biological implications will be presented elsewhere. Here, we focused mainly on the computational analysis. Additionally we propose a novel method to validate the cross-linked site assignment: by relaxing specificity of a known, selective cross-linker. For this purpose we used the Rim1 cross-linked dataset obtained using DSS.

## Methods

### Algorithm

The algorithm and the program workflow of XLPM are shown in Additional file 1. XLPM receives information of protein sequences, a spectra file in mgf format, digestion enzyme, and cross-linker, static and variable modifications of amino acids, missed cleavage level, precursor ion tolerance in ppm and fragment ion tolerance in Daltons. XLPM generate temporary database of cross-linked pairs from given protein sequences. First, amino acids that may be cross-linked are marked in the protein sequence. A list of digested fragment sequences is generated. A missed cleavage is forced. In other words, digested fragments having only one amino acid at the C terminal, which can be cross-linked, are removed from the list. The digested fragments devoid of any amino acids that can be cross-linked are also filtered out. A database of cross-linked pairs is generated from the list of digested fragments including the change of the mass due to any amino acid modification.

A file in a mascot generic format (mgf) is uploaded and read; data are extracted from it and parsed into precursor ions and fragment ions. The precursor ion masses are compared with the masses of cross-linked pairs in the database generated previously with the user defined error tolerance. Each matched cross-linked product is further analyzed using its MS/MS fragmentation pattern and scored.

### b-y filter and analysis of fragment ion spectra

**Figure 1:**
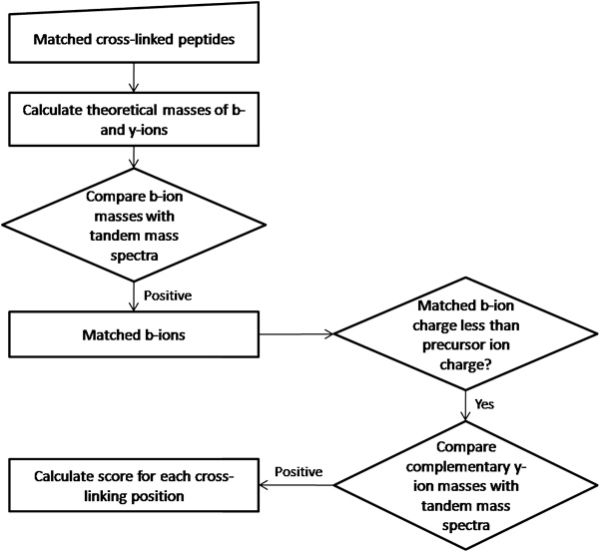
XLPM implements a unique filter based on high probability to observe a complimentary y-ion, given a b-ion. For each candidate pair of cross-linked peptides and for each possible cross-link position theoretical masses of b and y ions are computed. Next, the candidate experimental spectrum is checked for the presence of b-ions. In every case when charge of b-ion is less than the charge of the precursor, the experimental spectrum is searched for the complementary y-ion.

XLPM considers b and y ions while analyzing MS/MS fragmentation pattern. Cross-linked amino acids carry an additional mass of cross-linker and the second fragment. Thus, it can be treated as a modification on that amino acid while calculating the theoretical masses of the b and y ions. The algorithm calculates the mass of each b or y ion with and without the cross-linked second fragment. The algorithm then compares all calculated b ions (with and without the cross-linked fragment) with the MS/MS spectra. Complementary y ions of matched b ions are then compared with MS/MS spectra (Figure 1).

### Workflow and implementation

**Figure 2:**
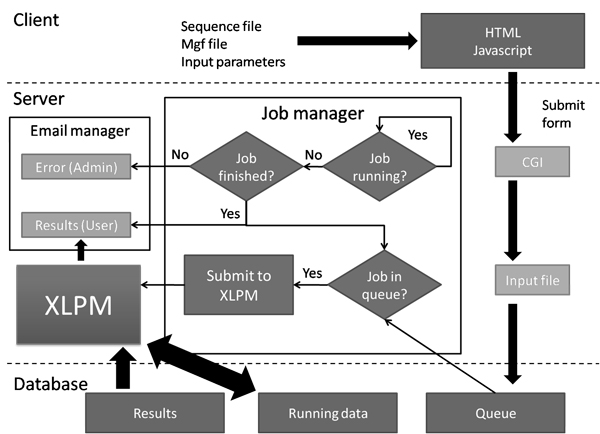
**The workflow of XLPM web server**. The job manager manages the submitted jobs. Once a completed job is detected by the job manager, it submits the next job in the queue to XLPM for the analysis. The results are mailed to the users and errors are mailed to the administrator, if any.

XLPM is a library of Perl scripts. All of the scripts are interdependent on one another. Each script serves a specific function in the course of the algorithm. Thus, a small modification in any of the scripts can tweak the algorithm to do a variety of analyses. The program uses a MySQL database to store the user input and the processed information at each stage of the analysis, which again aids in making the scripts efficient and flexible. XLPM can be accessed through a simple and intuitive web interface (http://binf-app.host.ualr.edu/~mihir/cgi-bin/xlpm.cgi) which allows the user to submit jobs to the system. A web form allows variables to be set and input files to be uploaded. Jobs are placed in a queue and upon completion results are emailed to the user. The workflow is depicted in Figure 2.

The database contains pre-compiled data of digestive enzymes, cross-linkers, modifications, and amino acids. A new enzyme, cross-linker or custom modification can easily be added to the database and used in subsequent analyses. The algorithm is compatible with digestive enzym cleaving at either end of one or more amino acids. The algorithm can also handle all kinds of amino acid modifications for which precise changes in the masses are known. XLPM is better than many existing tools, as it can accommodate specific, semi-specific and non-specific cross-linkers. The algorithm can also perform isotopic analysesusing^15^N-labled proteins.

### Scoring

The XLPM score is a representation of the proportion of matched b-ions and y-ions, using the following formula:

$\text{XLPM}\;\text{score}=\frac{{\text{N}}_{\text{mb}}}{{\text{N}}_{\text{tb}}}+\frac{{\text{N}}_{\text{my}}}{{\text{N}}_{\text{ty}}}\frac{1}{{\text{R}}_{\text{yb}}}$.

N_mb _and N_my _are numbers of theoretical b ions and y ions matched to the CID spectrum, respectively. N_tb _and N_ty _are numbers of total possible b ions and y ions, respectively. R_yb _is the median ratio of complementary y ions to b ions for the charge of the precursor ion as calculated from NIST the data, described in the previous segment.

### Materials

The following materials were purchased from Thermo Fisher Scientific or its subsidiaries: HPLC-grade acetonitrile, formic acid, HEPES, Tris, NaCl, EDTA, MgCl2, SDS, KOH, β-mercaptoethanol, acrylamide, bisacrylamide, formamide, xylene cyanol, bromphenol blue, urea, glycerol, SDA cross-linker, formaldehyde, DSS, Gel-Code blue stain, and Zeba-Spin Desalting columns for buffer exchange.

### Recombinant proteins

Recombinant helicase domain of Pif1, full-length Pif1, and Rim1-C-terminal 6xHis proteins used in this work were purified as described in [14] K29A Rim1-C-terminal 6xHis mutant was purified from *E. Coli*, using the same isolation protocols as for wild-type Rim1, established in [1]. Throughout the article, we call Rim1-C-terminal 6xHis as wild type Rim1, and K29A Rim1-C-terminal -6xHis as K29A Rim1. The proteins without 6xHis tag as labeled as "no-tag", when appropriate.

### ^15^N-labeled Rim1

To metabolically label Rim1, *E. Coli *expressing Rim1 was grown on media prepared with ^15^N-ammonium chloride as the sole source of nitrogen [15]. Next, the 15N-labeled Rim1 was purified according to the previously established protocol [16].

### Cross-linking reactions using DSS

To the 2X stock of Rim1 protein (6 µM of Rim1 tetramer), was added equal volume of 5 mM DSS in a cross-linking reaction buffer. Final concentrations of the reagents in the reaction mix were: 25 mM HEPES, 100 mMNaCl, 10% Glycerol, 3 µM of Rim1 tetramer, 2.5 mM DSS, pH = 7.5. The reaction was incubated at room temperature for 40 min, with mild agitation. Next, the quenching buffer (Tris, pH 8) was added to the final concentration of 50 mM. Using Zeba Spin Desalting columns (Thermo Fisher), the buffer was exchanged into 25 mM HEPES, 100 mMNaCl, pH 7.5. 90% protein recovery was verified by Bradford assay. The mixture was boiled in SDS loading buffer, and the products were resolved on 5-15% gradient SDS-PAGE.

### Cross-linking reactions using SDA

Prior to the cross-linking experiments, the proteins were thawed on ice, and using Zeba Spin Desalting columns, the storage buffer was exchanged to the cross-linking reaction buffer (25 mM HEPES, 100 mM NaCl, 10% Glycerol, pH 7.5). In all of the cross-linking reactions, the final concentrations of Rim1 (tetramer) were 3 µM. To the 2X stock of Rim1 protein in the cross-linking buffer (6 µM of Rim1), SDA stock solution in DMSO was added (50 µM SDA and 0.5% DMSO final concentrations). The NHS reaction was carried out at 25 °C for 30 min followed by the addition of 1M Tris, pH 8.0 (50 mM final Tris) and the additional incubation at 25 °C for 5 min. Next, to remove the unreacted cross-linker, the buffer was exchanged with fresh cross-linking buffer using the Zeba Spin Desalting columns. A 90-95% percent protein recovery was verified by Bradford assay. For the second step of the SDA-cross-linking reaction, an equal volume of the 2X Rim1 protein or 2X Pif1 protein, to be reacted, was added to the NHS-reacted mix. UV-cross-linking was performed in a Strata linker 1800 using coated bulbs (365 nm emission maximum) at a 2 cm distance from the bulbs in black, flat-bottomed 96-well plates. 4X SDS buffer was added to the reactions and the reaction products were resolved on a5-15% gradient SDS-PAGE. The cross-linked band was excised, in-gel digested and analyzed by the NanoLC-LTQ-Orbitrap-Velos mass spectrometry, as described below.

### NanoLC-LTQ-Orbitrap mass spectrometry, Identification of cross-linked peptides and Mass spectrometry analysis

Gel slices were destained in 50% methanol, 100 mM ammonium bicarbonate, followed by reduction in 10 mM Tris[2-carboxyethyl] phosphine and alkylation in 50 mM iodoacetamide. Gel slices were then dehydrated in acetonitrile, followed by addition of 100 ng porcine trypsin in 100 mM ammonium bicarbonate and incubation at 37°C for 12-16 hours. Peptide products were then acidified in 0.1% formic acid. Tryptic peptides were separated by reverse phase Jupiter Proteo resin (Phenomenex) on a 100 × 0.075 mm column using a nano Acquity UPLC system (Waters). Peptides were eluted using a 30 min gradient from 98:2 to 40:60 buffers A:B ratio. [Buffer A = 0.1% formic acid, 0.05% acetonitrile; buffer B = 0.1% formic acid, 75% acetonitrile.] Eluted peptides were ionized by electrospray (1.9 kV) followed by MS/MS analysis using collision induced dissociation on an LTQ Orbitrap Velos mass spectrometer (Thermo).

To detect the cross-linked peptides, an LTQ-Orbitrap-Velos was used with the following parameters: 1) the three most intense precursors were subjected to MS/MS analysis - each of the precursors was analyzed consecutively by HCD (7,500 resolution), CID with the analysis of the fragments in OrbiTrap (FT-CID 7,500 resolution), and regular CID; 2) each sample was run twice, the first time allowing all of the charges, and the second time allowing only precursors of charge 4 and higher to be fragmented; 3) the precursor selection intensity threshold was set to 5000; 4) for the ^15^N-labeled samples, only the HCD fragmentation of the 10 most intense precursor ions at 7,500 resolution was used.

### Data analysis and peak lists preparation

Peaks lists in a mascot generic format were generated from the raw files using Proteo Wizard tools. The same sets of parameters were used for both XLPM and StavroX. An input file with tags and values was made for XLPM. To evaluate a possible impact of charge deconvolution of fragment spectra we used spectral refinement function available in the PEAKS v7 software suite (Bioinformatics Solutions Inc.). The spectral deconvolution did increase the scores obtained through StavroX by annotating more highly-charge fragments, while no improvement was observed in the case of XLPM, because XLPM already had most of the highly charged fragments (*data not shown*).

### Protein structure visualization

To construct, manipulate, and visualize the X-ray structure we used PyMOL (The PyMOL Molecular Graphics System, Version 1.3.0.0 Schrödinger, and LLC).

## Results

### XLPM web interface

**Figure 3:**
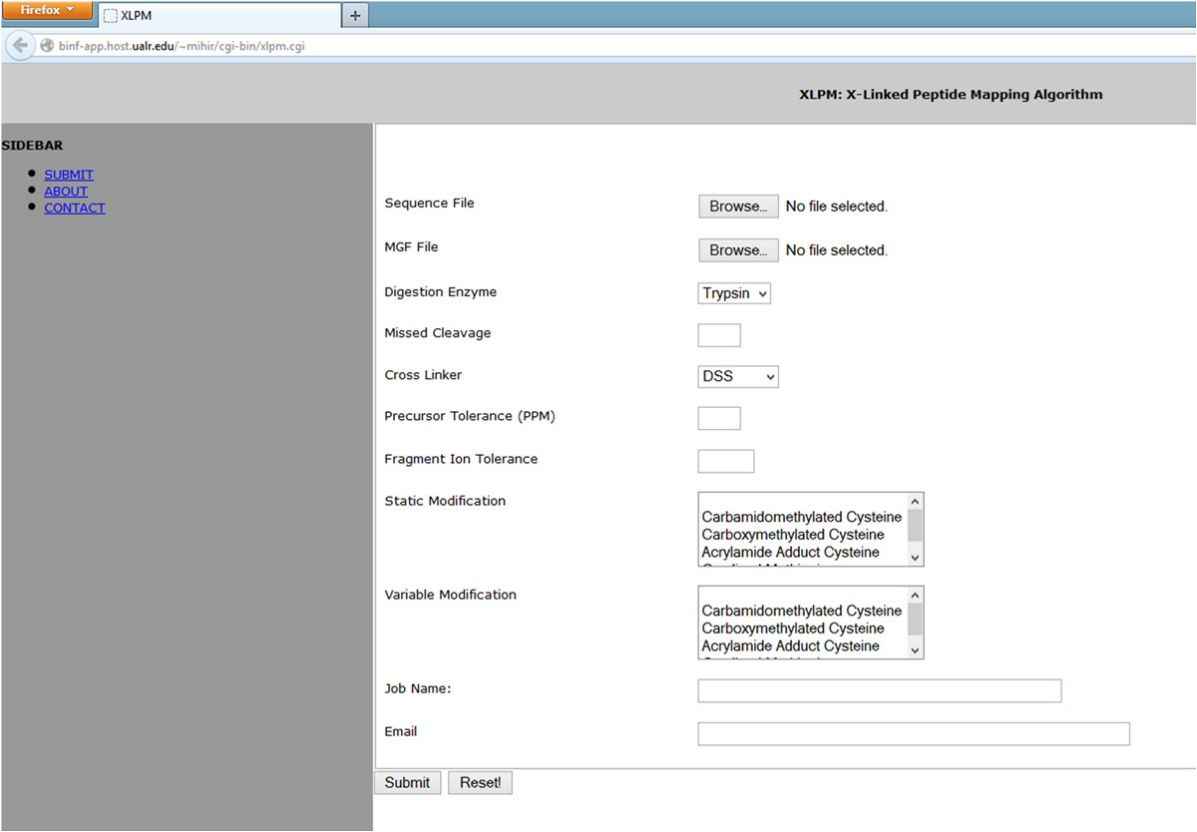
**Job submission web-page for XLPM**. The screen-shot of the XLPM job submission page, available at http://binf-app.host.ualr.edu/~mihir/cgi-bin/xlpm.cgi

XLPM is implemented both as a stand-alone program, with the source code freely available, and an online tool. The online interface is user friendly and very straightforward (Figure 3).

### Validation of the b-y filter on a set of linear peptides

The algorithm, comparing MS/MS spectra of precursor ion with all b ions and complementary y ions, uses b-y ion filter and is based on the observation, "Detection of a particular b ion with the charge less than that of the precursor implies a high probability of detection of the complementary y-ion with the remaining charge." As the amino acid sequences of proteins and the details of cross-linker are known, the algorithm can analyse the results without matching the entire MS/MS spectra. Matching all b ions and complementary y ions reduces the work of the algorithm considerably, making it fast and efficient.

We analysed the NIST (National Institute of Standard and Technology) (http://peptide.nist.gov/) library of annotated ion trap spectra to validate the b-y filter. For an MS/MS spectrum of each precursor, b-ions having charges less than that of the corresponding precursor ion were identified, and the spectrum was then scanned for the complementary y ions with the remaining charge. The ratio of the number of such y ions to the number of b ions was calculated for the spectrum of each precursor ion. All the ratios were plotted separately for each of the charges; two, three, four, five and higher.

**Figure 4:**
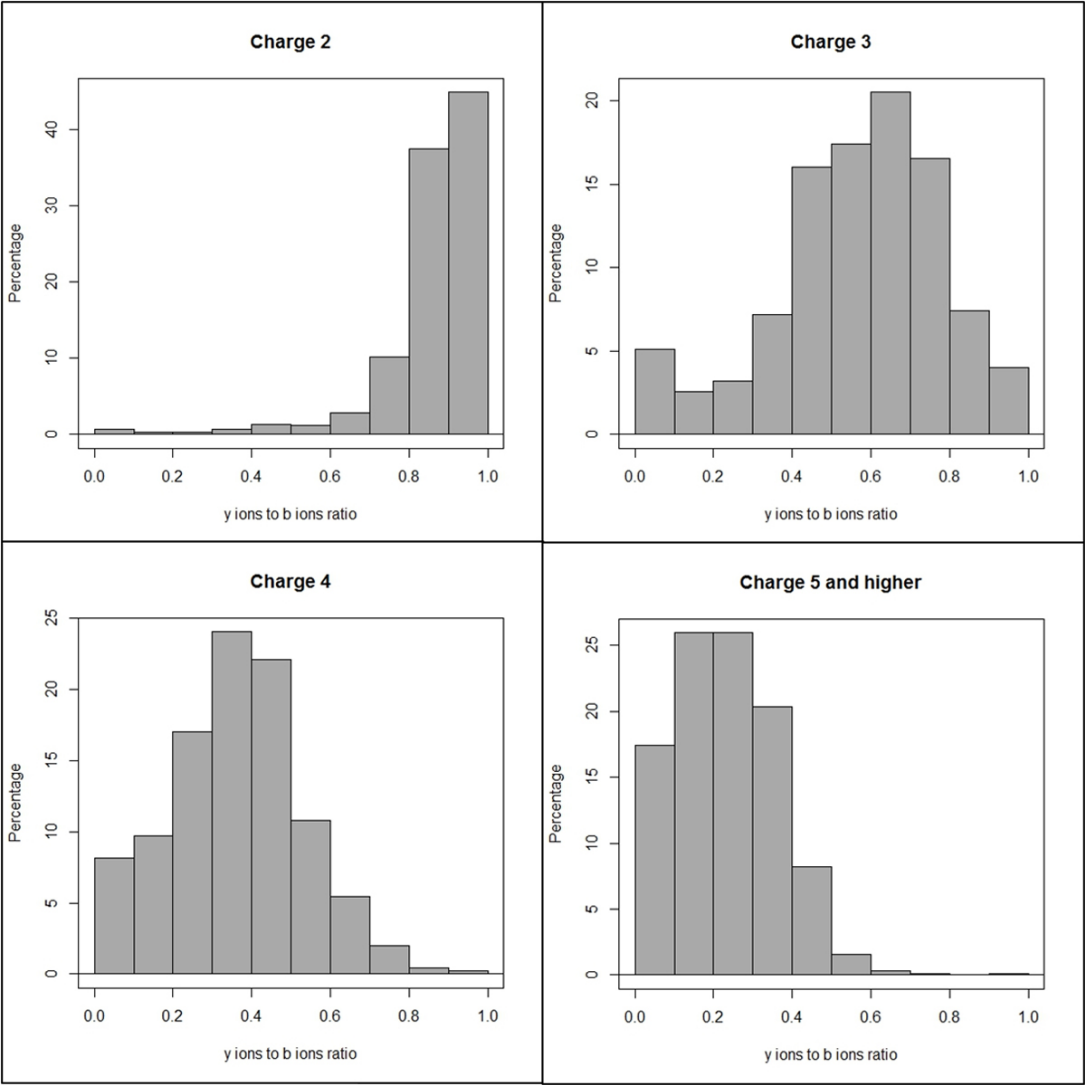
**Histograms of complementary y ions with remaining charge to b ions ratio derived from NIST data set of annotated peptides**. The number in the chart is the median value of the distribution.

The graphs in Figure 4 show the histograms of the above mentioned ratios for precursors with charges two, three, four, five and higher. As the distributions of the ratios are non-zero, the probability of the complementary y ion with the remaining charge being present when a b ion is present is non-zero, which supports the observation. In addition, the histograms show a negative correlation with the charge. As the charge increases, the distribution shifts to the left (the ratio moves towards zero).

### Mapping of the Rim1 tetramer DSS cross-links

**Figure 5:**
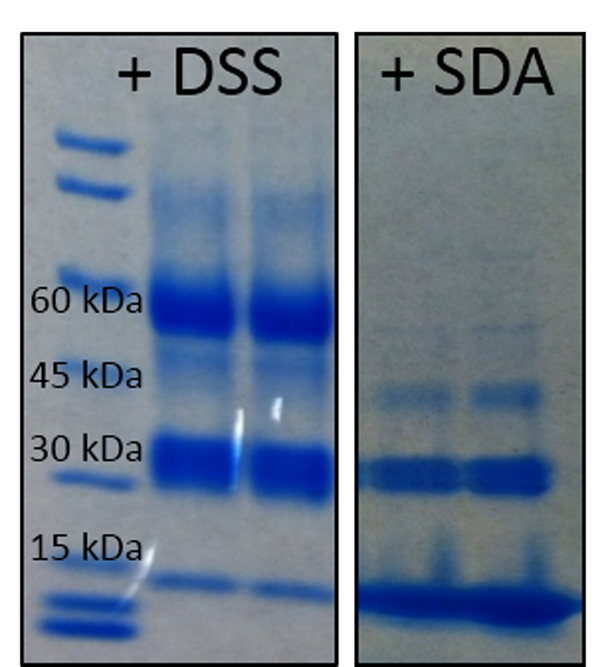
**Products of cross-linked Rim1 tetramer resolved on SDS-PAGE**. *Left panel *- results of the DSS cross-linking. *Right panel *- results of SDA cross-linking. 60kDA band corresponds to fully assembled Rim1 tetramer. 45 kDa band - to trimer, 30 kDa - to dimer, and the 15kDa band - to monomer.

**Figure 6:**
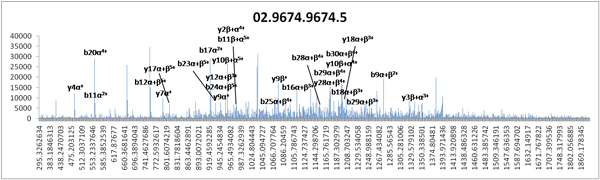
**A representative MS/MS spectra derived from DSS-cross-linked peptide annotated by XLPM**. The header in the graph depicts the scan number in the spectra. Fragment sequence 1: KGALVYVEADAANYVFERDDGSKGTTLSLVQK. Fragment sequence 2: YLKYSIASQPRR. Mass to charge: 1013.544006. Charge: 5+.

Rim1 tetramer was cross-linked using DSS, a 11.4A-long cross-linker, cross-linking primary amino groups. Figure 5, ***left panel ***shows the migration of protein bands corresponding to Rim1 tetramer (~60 kDa), Rim1 dimer (~30 kDa), and monomer (~15 kDa) on the gel. The mass spectra were analysed using XLPM and StavroX and the feasibility of the cross-links was assessed by mapping of the cross-links onto the available Rim1 X-ray structure. The monomer band did not yield any cross-links. The dimer and tetramer bands showed a similar pattern of interactions. Mainly, four lysines - 29, 64, 86, 105 - were found to be involved in various cross-links. Furthermore, lysine 105 cross-linked with lysine 64 was the highest scoring cross-linked pair in the dimer. A cross-link between this pair was also present in the tetramer band. Cross-linking of lysine 29 with lysine 86 was also a prominent interaction found by both XLPM and StavroX. False discovery rate was controlled by search against database based of reverse Rim1 sequence. With the XLPM found 33 and 18 cross-linked pairs from tetramer and dimer of Rim1 with better than 5% FDR [17]. Similar cross-linking sites were found by StavroX. Tables 1, 2, 3, 4 provide summary of StavroX and XLPM-identified cross-links. The major difference between StavroX and XLPM results were cross-linking between C terminal beta sheets in StavroX. However, the rigid beta sheet structure at C terminal in the Rim1 makes it improbable site for DSS cross-linking, DSS being a cross-linker with long spacer arm (11.4 Ă) (Additional file 2A), making cross-linking between any pair of lysines 95,101 and 104 unlikely. In addition, four or higher charge is expected of cross-linked species. Most of the cross-link pairs involving lysines 95, 101, 104 identified by StavroX have charge less than four, decreasing the feasibility of these cross-links (as we expect such species to ionize at charge 4+ and higher). While, all the cross-linked pairs identified by XLPM have been of charge four and higher. Figure 6 shows one of the fragment spectra, from DSS-cross-linked dataset, annotated by XLPM. The x-ray crystal structure for Rim1 is solved for residues 2-104. Additional file 3 depicts the highest scoring cross-link determined by XLPM between lysines 64 and lysines 105. The red circle shows possible position of lysine 105 (as this lysine is absent from in the crystal structure). Additional file 4 shows another predicted cross-link pair of lysine 29 and lysine 86. Furthermore, a cross-link between lysine 105 and N-terminal is also plausible as predicted by both XLPM and StavroX.

**Table 1:** StavroX results for DSS cross-linking of Rim1 tetramer

Score	m/z	Charge	Precursor mass	Calculated mass	PPM	Sequence 1	Sequence 2	Cross-linked residues
233	542.5646	4	2167.237	2167.233	1.48	[DINLLKNGK]	[DIDLLKNGK]	101-101
228	867.1219	3	2599.351	2599.34	4.25	[DGKK]	[KGALVYVEADAANYVFER]	104-64
228	599.3096	3	1795.914	1795.915	-0.16	{mDFSK]	[DINLLKDGK]	1-101
222	620.3467	4	2478.365	2478.36	1.97	[DINLLKNGK]	[YLKYSIASEPR]	101-29
218	542.5644	4	2167.236	2167.233	1.26	[DINLLKNGK]	[DIDLLKNGK]	101-101
190	533.9707	3	1599.898	1599.895	1.47	[NGKK]	[DIDLLKDGK]	104-101
182	971.1868	3	2911.546	2911.541	1.59	[YLKYSIASQPR]	[DDGSKGTTLSLVQK]	29-86
159	759.7696	5	3794.819	3794.812	1.96	[DINLLKNGK]	[KLEDAEGQEDAASSELEHHHHHH}	101-105
149	1012.199	3	3034.581	3034.579	0.62	[DDGSKGTTLSLVQK]	[DDGSKGTTLSLVQK]	86-86
134	1199.317	4	4794.246	4794.257	-2.19	[KGALVYVEADAANYVFER]	[KLEDAEGQENAASSELEHHHHHH}	64-105
132	699.6585	4	2795.612	2795.613	-0.23	[DINLLKNGK]	[GTTLSLVEKDINLLK]	101-95
129	900.9684	4	3600.852	3600.843	2.37	[DDGSKGTTLSLVQK]	[KGALVYVEADAANYVFER]	86-64
125	702.6923	3	2106.062	2106.058	2.2	{mDFSK]	[YLKYSIASQPR]	1 29
119	856.3835	4	3422.512	3422.509	0.96	{mDFSK]	[KLEDAEGQENAASSELEHHHHHH}	1-105
118	636.6761	3	1908.014	1908.015	-0.51	{MDFSK]	[DINLLKDGKK]	1-104
117	818.7658	6	4907.559	4907.547	2.43	[YLKYSIASQPR]	[KGALVYVEADAANYVFERDDGSKGTTLSLVQK]	29-64
115	808.4442	3	2423.318	2423.31	3.26	{mDFSK]	[GTTLSLVQKDINLLK]	1 95
115	792.6701	4	3167.659	3167.662	-1.17	[DIDLLKNGK]	[KGALVYVEADAANYVFER]	101-64
112	646.1035	5	3226.489	3226.49	-0.37	[DGKK]	[KLEDAEGQENAASSELEHHHHHH}	104-105
108	432.7513	4	1727.983	1727.99	-3.91	[DGKK]	[DIDLLKNGKK]	104-104
108	982.3183	5	4907.562	4907.547	3.09	[DIDLLKNGKK]	[MSIVGRIGSEFTEHTSANNNRYLKYSIASQPR]	104-29

The table shows only the highest scoring results for a cross-linked pair

**Table 2:** StavroX results for DSS cross-linking of Rim1 dimer

Score	m/z	Charge	Precursor mass	Calculated mass	PPM	Sequence 1	Sequence 2	Cross-linked residues
587	574.8339	4	2296.3136	2296.3122	0.6	[DINLLKDGK]	[DIDLLKNGKK]	101-101
243	533.9705	3	1599.8968	1599.8952	1.01	[NGKK]	[DIDLLKDGK]	104-101
236	866.7906	3	2598.3574	2598.3562	0.45	[NGKK]	[KGALVYVEADAANYVFER]	104-64
224	599.3107	3	1795.9176	1795.9146	1.68	{mDFSK]	[DIDLLKNGK]	1-101
201	633.309	6	3794.8185	3794.8117	1.78	[DINLLKNGK]	[KLEDAEGEENAASSELEHHHHHH}	101-105
187	971.1871	3	2911.5468	2911.5411	1.96	[YLKYSIASQPR]	[DDGSKGTTLSLVQK]	29-86
130	1012.1968	3	3034.576	3034.579	-1.01	[DDGSKGTTLSLVQK]	[DDGSKGTTLSLVQK]	86-86
114	856.3831	4	3422.5106	3422.5091	0.46	{mDFSK]	[KLEDAEGQENAASSELEHHHHHH}	1-105
113	792.6713	4	3167.664	3167.6623	0.52	[DINLLKNGK]	[KGALVYVEADAADYVFER]	101-64
113	636.6771	3	1908.0166	1908.0147	1.03	{MDFSK]	[DINLLKDGKK]	1-104
110	799.8846	6	4794.2715	4794.2567	3.07	[KGALVYVEADAANYVFER]	[KLEDAEGQENAASSELEHHHHHH}	64-105
108	650.5901	4	2599.3385	2599.3402	-0.66	[DGKK]	[KGALVYVEADAANYVFER]	105-64
102	821.4157	5	4103.0494	4103.0455	0.96	[DDGSKGTTLSLVQK]	[KGALVYVEADAANYVFERDDGSK]	86-64

The table shows only the highest scoring results for a cross-linked pair

**Table 3:** XLPM results for DSS cross-linking of Rim1 tetramer

m/z	Sequence 1	Sequence 2	Calculated mass	Precursor mass	Charge	XLPM score	Cross-linked residue
799.8825073	kLEDAEGQENAASSELEHHHHHH	kGALVYVEADAANYVFER	4793.26531	4793.248047	6	1.355730948	105-64
1013.544006	kGALVYVEADAANYVFERDDGSkGTTLSLVQk	YLkYSIASQPRR	5062.65627	5062.680664	5	1.341551127	29-86
568.5924683	kLEDAEGQENAASSELEHHHHHH	mDFSk	3405.52264	3405.507813	6	1.340157423	105-1
720.9754028	kGALVYVEADAANYVFER	DDGSkGTTLSLVQk	3599.85173	3599.837891	5	1.25584922	64-86
870.2092896	kGALVYVEADAANYVFER	YLkYSIASQPR	3476.81381	3476.805908	4	1.200043431	64-29

The table shows only the highest scoring results for a cross-linked pair

**Table 4:** XLPM results for DSS cross-linking of Rim1 dimer

m/z	Sequence 1	Sequence 2	Calculated mass	Precursor mass	Charge	XLPM score	Cross-linked residues
799.8850098	kLEDAEGQENAASSELEHHHHHH	kGALVYVEADAANYVFER	4793.26531	4793.263184	6	1.301676894	105-64
728.6427002	DDGSkGTTLSLVQk	YLkYSIASQPR	2910.54962	2910.539551	4	1.105475415	86-29
900.9683838	kGALVYVEADAANYVFER	DDGSkGTTLSLVQk	3599.85173	3599.842285	4	1.094423763	64-86

The table shows only the highest scoring results for a cross-linked pair

**Figure 7:**
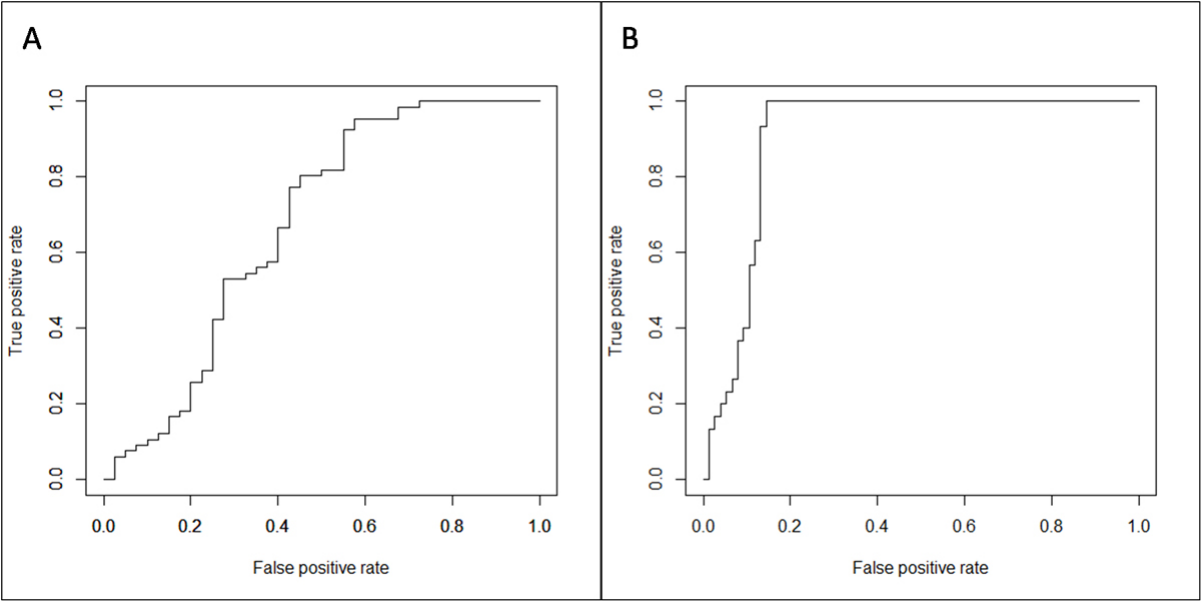
**ROC curves of the DSS analysis**. The specificity of DSS at the second end was removed. *A*, No score cutoff, AUC: 0.6727.*B*, Score cutoff at 5% FDR, AUC: 0.9066.

While the false discovery rate of finding a cross-linked peptide pairs can be estimated using decoy databases, it is not apparent how to estimate the false positive rate of the cross-link site assignment within already identified peptide pair. In this work, for the first time, we proposed the relaxed specificity approach: in analysing the false positive rate of cross-linked site assignment a specific cross-linker (e.g. Lys-to-Lys cross-linker DSS) is treated as non-specific (e.g. Lys-to-any) by XLPM. Any sites of cross-linked attachment outside the range of known cross-linker's specificity found in such way are considered false-positives. Figure shows such ROC curves. Figure A shows ROC curve when no filter is applied on the XLPM score, considering all positive scored results as positives. Figure B shows ROC curve for the results at 5% FDR (Figure 7).

### Rim1 tetramer cross-linking using the non-specific cross-linker SDA

SDA cross-linker connects lysine at one end to any amino acid at the other. We developed XLPM to include non-specific analysis with no limitations. Rim1 tetramer was cross-linked using the SDA (Figure 5, ***right panel*) **and the cross-linked products were analysed using XLPM. XLPM successfully identified nine cross-linked candidates in each of the two bands. This result illustrates the ability of XLPM not only to identify the peptides cross-linked using a non-specific cross-linker, but also XLPM's ability to distinguish between the sites of cross-linking. Two confident sites were identified by XLPM from the SDA-cross-linked spectra: N-terminus MDFSK to YLKSIAPQR peptide (Additional file 5**)**, and N-terminus MDFSK to C-terminal peptide LEADAEGQENAASSEHHHHHH.

### Identification of interaction between Rim1 tetramers using 15N-labeling

**Figure 8:**
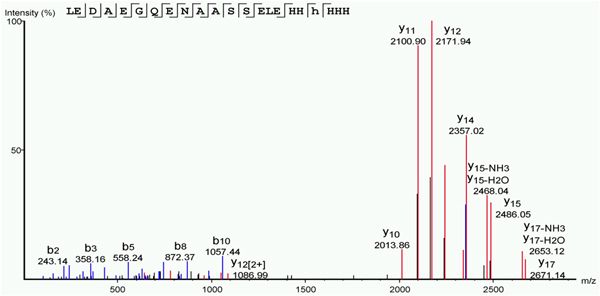
**N-terminus to C-terminus interaction occurs between different subunits of Rim1**. The spectra consisting of 14N-labeled C-terminus and 15N-labeled N-terminus is shown. After identification with XLPM, the result was verified independently with PEAKS v7, encoding 15N-labeled peptide, MFDSK as variable modification. The spectrum as preprocessed and annotate by PEAKS is shown.

To investigate a possibility of Rim1 tetramers interacting with each other, we prepared separately ^14^N-labeled Rim1 tetramer and ^15^N-labeled Rim1 tetramer, mixed them at 1:1 ratio and subjected to the cross-linking analysis using SDA and XLPM. Multiple spectra were identified corresponding to the N-terminal MDFSK peptide cross-linked to C-terminal LEDAEGQENAASSEHHHHHH peptide. Cross-linked species, where one peptide chain is 15N-labeled and the other is 14N-labeled were clearly visible. Figure 8 shows the most visible 15N-14N interaction, verified and annotated by PEAKS v7 software suit. The spectra were analyzed in PEAKS with MDFSK peptide specified as variable modification to validate the results of XLPM. In contrast, the other site of SDA-cross-ling MDFSK to YLKSAIPQR did not show mixed 14N-15N cross-linked - only 14N-14N and 15N-15N species were observed. These results clearly indicate that the N-terminus to C-terminus SDA-derived cross-link arises due to interaction between different subunits of Rim1, either from direct interactions between Rim1 tetramers, or from dissociation and reassembly of Rim1 tetramers.

## Discussion

### Novel features of XLPM compared to other algorithms for cross-link analysis

The b-y filter implemented in the current version of XLPM, makes the cross-link identification considerably faster compared to existing algorithms. This is because only a sub-set of fragment ions is calculated in theoretical spectra and considered for identification. Even for linear peptides, using the analysis of the set of NIST MS/MS spectra, we showed that there is a high correlation between the presence of a b-ion and the presence of the complementary y-ion in the same spectrum. This is in line with the observations by Frank *et. al*. that b and y ions are highly correlated in doubly charged peptides [18]. Furthermore, Tabb *et. al*.[19] noted that y-ions are usually higher in intensity than the complementary b ions, in the case of tryptic peptides. Therefore, if a b-ion is observed in an MS/MS spectrum, it is highly likely that the complimentary y-ion will also be observed. While it is certainly true that having a complimentary set of b and y ions is not a requirement for peptide identification, what is likely - is that when such condition occurs it indicates that the MS/MS spectrum is of very high quality, leading to a high-scoring identification. The b-y filter, therefore, could be thought as a quality filter. Our analysis of NIST data suggests that even a regular data-base search of a proteomics dataset may benefit from such a filter. We also expect that for the non-linear, cross-linked peptides, the b-y filter should perform just as well as for linear peptides. Indeed, a fragmentation spectrum of a regular linear peptide precursor in a typical experiment using collisional excitation, conforms to a "mobile proton model": when peptide is collisionally excited, a proton from its protonated N-terminus is free to travel along the peptide backbone and to form intermediates with protonated amide nitrogens, with protons on the side chains of arginines and lysins (e,g, in the case of tryptic ends) remaining immobile[20]. For the most type of cross-linkers it is safe to assume that mobile protons will not jump through the cross-link and will remain localized to their original peptide chains. We should therefore, expect that the fragmentation spectrum of a typical charge 4+ cross-linked pair of tryptic peptides will resemble a sum of fragment spectra of individual charge 2+ peptides, as opposed to a fragment spectrum of a charge 4+ linear peptide. The b-y filter could be easily modified, if needed, to accommodate different types of experiments - e.g. when intensity of b-ions is enhanced or suppressed through modification of peptide N-termini. It can also be easily implemented as a c-z filter for spectra obtained using electron transfer dissociation; however, we have not yet tested its effectiveness in this setting due to the lack of standard annotated datasets of ETD spectra.

XLPM also does better job at annotating higher-charged fragments compared to other cross-linked identification algorithms. Partially, this is due to b-y filter, as the XLPM searches for a complimentary y-ion having a charge such, that the sum of charges is equal to the charge of the precursor. In fact we have not found much improvement in XLPM performance upon deconvolution of highly-charged fragments into 1+ fragments (*data not shown*).

### False positive rate of cross-linked site identification

XLPM performs well, without sacrificing much of the analysis speed, when one of the cross-linker's functionality is non-specific and cross-linking at any amino-acid is possible. Finding cross-linked peptides in mass spectra is similar to finding a needle in a haystack. The mass spectra contain majority of non cross-linked peptides and a small number of cross-linked peptides. Target-decoy method of finding FDR has been widely acceptable in protein mass spectrometry data analysis. However, this method should be used with extra caution in cross-linking mass spectra analysis. As the expected correct matches of cross-linked peptides with the mass spectra are small, few hits from decoy may shoot up the FDR significantly. By putting stringent FDR requirements may eliminate some of the matches as false negatives.

In case of XLPM, we have used target-decoy method to calculate FDR and successfully validated the results. However, target and decoy sequences were analyzed separately, which may have boosted the score of some decoy sequences. In creating the database from protein sequences, XLPM removes database entries that do not contain any site of cross-linking, contains only one possible site of cross-linking at the N terminal of the digested fragment (forcing the miss cleavage), and digested fragments less than three amino acids. These filters decrease the difference in the characteristics of the target and decoy database. Thus, use of FDR requires caution in case of cross-linking analysis.

To validate XLPM's effectiveness in cross-linking assignment, we plotted the ROC curves with relaxed specificity of cross-linker at the second end. We plotted the ROC curves without any cut-off and 5% FDR. With the improvement in FDR, the area under curve for the ROC curve was also increased. Thus, relaxed specificity analysis can be an effective method for validation of cross-linking assignment and its usefulness at least as an auxiliary tool to the target-decoy method can not be ruled out.

## Conclusions

XLPM is a fast and reliable algorithm for the identification of cross-linked peptide pairs. In XLPM we implemented, validated, and demonstrated utility of the unique spectral filter, which is based on the expected ratio of y-to-b ions in the fragment spectra. Furthermore, XLPM performs well in identification of the specific sites of the cross-linking, as demonstrated by the relaxed specificity ROC analysis.

## Competing interests

The authors declare that they have no competing interests.

## Authors' contributions

Mihir Jaiswal and Nathaniel Mark Crabtree wrote the bulk of the code. Mihir Jaiswal wrote the manuscript, supervised XLPM development, performed the computational analyses and validations. Mihir Jaiswal also proposed and validated the y-to-b ratio filter for the purpose of the cross-link analysis. Michael A. Bauer participated in the web-site development and the implementation of the online access. Roger Hall consulted the XLPM team and provided additional software development support. Boris Zybailov performed the cross-linking experiments and proteomics analysis of cross-linked products. Boris Zybailov and Kevin Raney designed the study and participated in the data analysis and interpretation.

## Funding

The work presented in this paper is supported by NIH Grant R01 GM098922 (to Kevin Raney), and by a UAMS Cancer Pilot Grant (to Boris Zybailov), the Arkansas IDeA Network for Biomedical Research Excellence P20GM103429 (to the UAMS Proteomics core), the UA Center for Protein Structure and Function (P30GM103450), and the UAMS Center for Microbial Pathogenesis and Host Inflammatory Responses (P20GM103625), Arkansas Tobacco Settlement grant and the INBRE grants NCRR (5P20RR016460-11) and NIGMS (8 P20 GM103429-11) from NIH. We acknowledge NIH R01 grant GM098922 for the funding of the publication of this article.

## Availability

XLPM is available to use on our web server at http://binf-app.host.ualr.edu/~mihir/cgi-bin/xlpm.cgi. Web server has limited options. However, if a user wants to use some other digestion enzyme, cross-linker or amino acids modifications, the user can email us with the details and we will update the web server to include the additional enzyme, cross-linker or amino acid modification. We also provide Perl code and MySQL dumps of XLPM upon request. One can customize the scripts and/or MySQL database to perform specific analysis. The code works with wide variety of enzymes, cross-linkers and amino acid modifications.

## Supplementary Material

Additional file 1 The XLPM algorithm to generate matched cross-link products. Click here for file

Additional file 2 Cross-links identified by XLPM mapped to the Rim1 X-ray structure. X-ray crystal structure of rim1 tetramer. The red color residues are C-terminal beta sheet of the DNA binding domain with three lysines - 95, 101, 104. The dense packing of atoms of the residues at C terminal makes DSS cross-linking in the region improbable. Click here for file

Additional file 3 Cross-link between lysine 64 and lysine 105 as identified by XLPM mapped to the Rim1 X-ray structure. Lysine 64 and Lysine 105 residue identified as cross-linked by DSS. Both residues are in flexible loop and likely candidate pair for DSS cross-linking as identified by XLPM. The red sphere shows lysine 64 while the red circle shows estimated position of the lysine 105 (the structure is not solved beyond 104-th residue). Click here for file

Additional file 4 Cross-link between lysine 29 and lysine 86 as identified by XLPM mapped to the Rim1 X-ray structure. Click here for file

Additional file 5 Cross-link between N-terminal and residues 27-38 peptide as identified by XLPM mapped to the Rim1 X-ray structure. Cross-linking between N-terminal and 27-38 peptide identified from by XLPM from SDA cross-linked Rim1 mass spectra. The red colored residues (27-38) are in vicinity of the blue colored N terminal. Click here for file
