# Supplementary figures and images for: XLPM: efficient algorithm for the analysis of protein-protein contacts using chemical cross-linking mass spectrometry
Source: BMC Bioinformatics. 2014 Oct 21;15(Suppl 11):S16. doi: 10.1186/1471-2105-15-S11-S16 (PMC4251045; doi:10.1186/1471-2105-15-S11-S16)

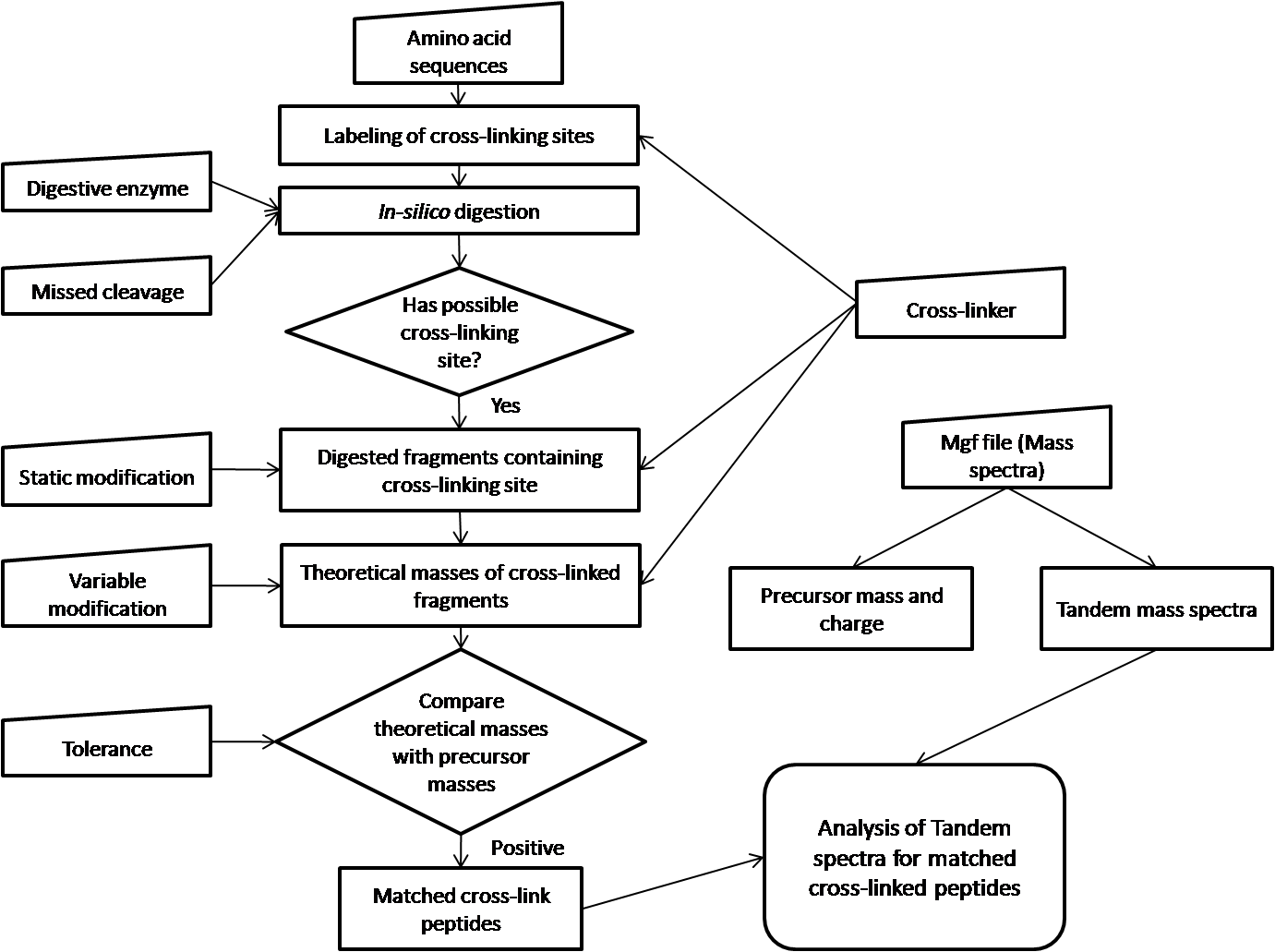

Supplement: Additional file 1 — The XLPM algorithm to generate matched cross-link products. [file 1471-2105-15-S11-S16-S1.png]

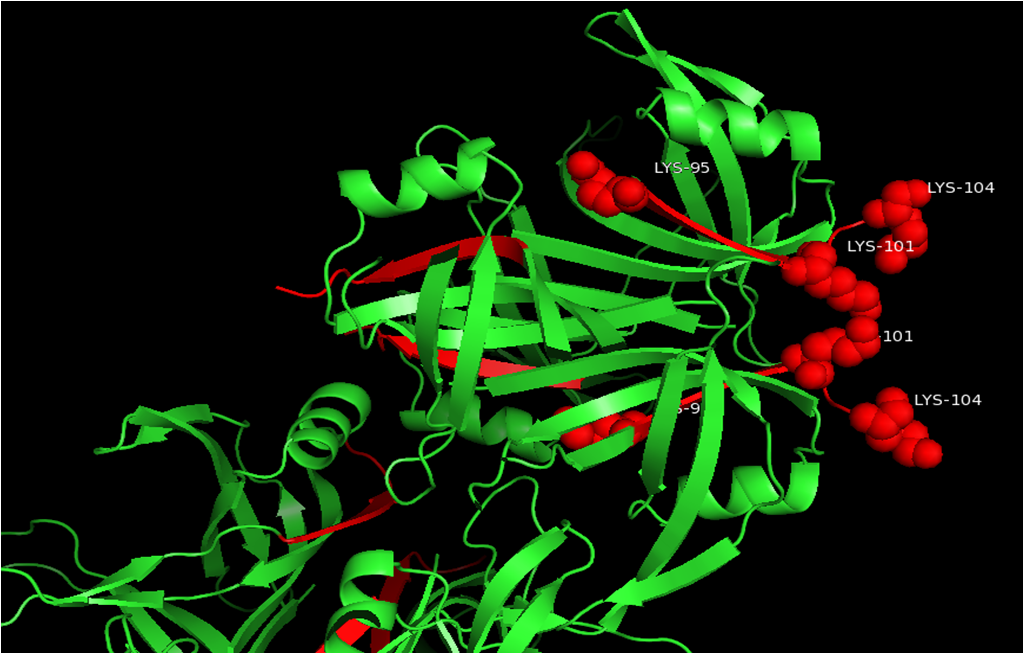

Supplement: Additional file 2 — Cross-links identified by XLPM mapped to the Rim1 X-ray structure. X-ray crystal structure of rim1 tetramer. The red color residues are C-terminal beta sheet of the DNA binding domain with three lysines - 95, 101, 104. The dense packing of atoms of the residues at C terminal makes DSS cross-linking in the region improbable. [file 1471-2105-15-S11-S16-S2.png]

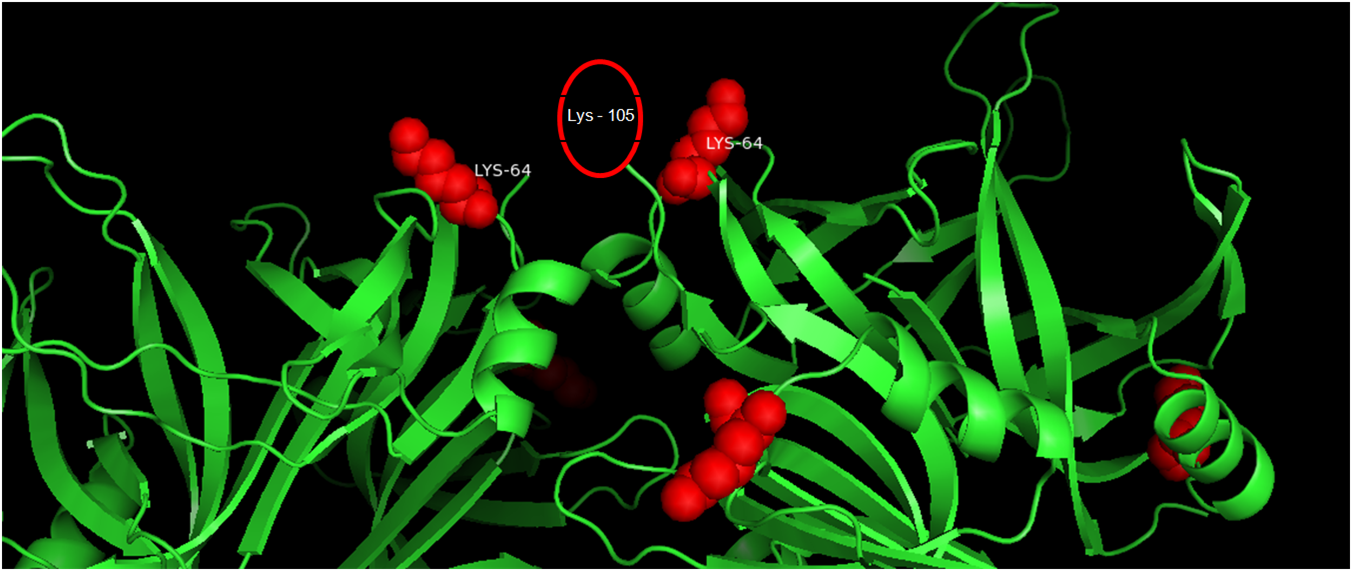

Supplement: Additional file 3 — Cross-link between lysine 64 and lysine 105 as identified by XLPM mapped to the Rim1 X-ray structure. Lysine 64 and Lysine 105 residue identified as cross-linked by DSS. Both residues are in flexible loop and likely candidate pair for DSS cross-linking as identified by XLPM. The red sphere shows lysine 64 while the red circle shows estimated position of the lysine 105 (the structure is not solved beyond 104-th residue). [file 1471-2105-15-S11-S16-S3.png]

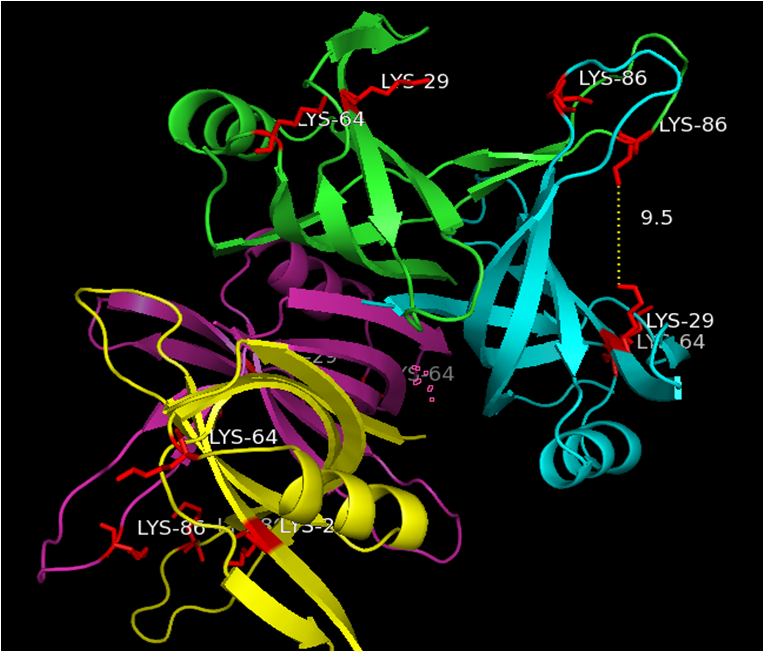

Supplement: Additional file 4 — Cross-link between lysine 29 and lysine 86 as identified by XLPM mapped to the Rim1 X-ray structure. [file 1471-2105-15-S11-S16-S4.png]

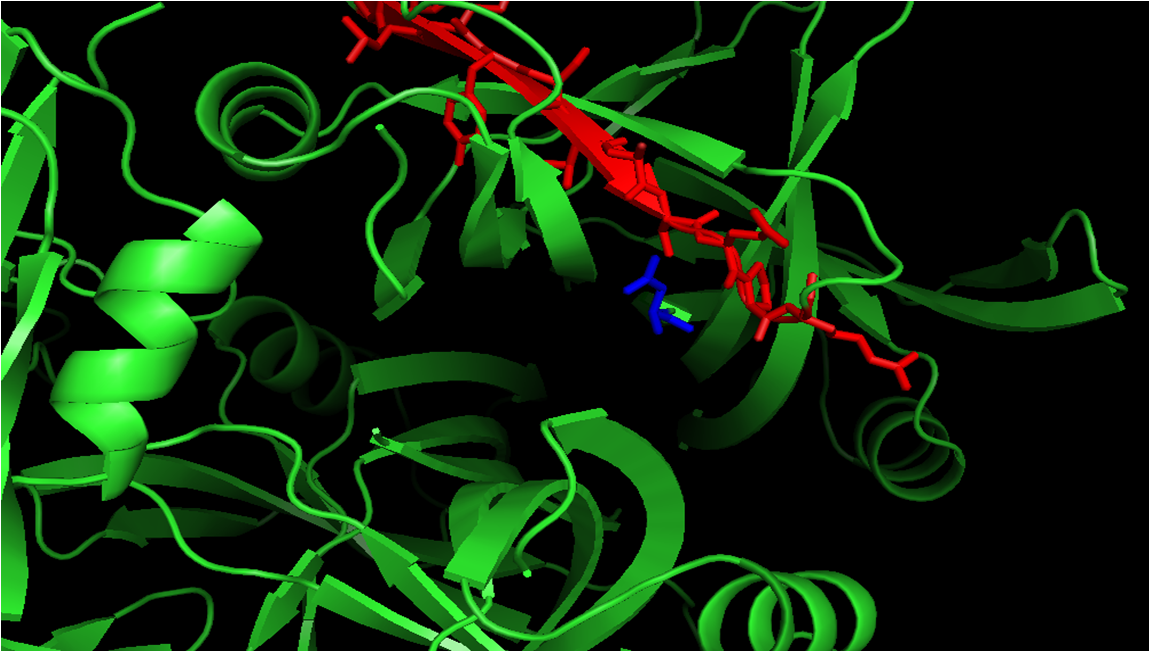

Supplement: Additional file 5 — Cross-link between N-terminal and residues 27-38 peptide as identified by XLPM mapped to the Rim1 X-ray structure. Cross-linking between N-terminal and 27-38 peptide identified from by XLPM from SDA cross-linked Rim1 mass spectra. The red colored residues (27-38) are in vicinity of the blue colored N terminal. [file 1471-2105-15-S11-S16-S5.png]
